# Empowerment and quality of life through cancer prevention in people experiencing homelessness: evaluating the Health Navigator Model

**DOI:** 10.1186/s12955-026-02588-8

**Published:** 2026-07-19

**Authors:** Tobias Fragner, Tamara Alhambra-Borrás, Ascensión Doñate-Martínez, Vicent Blanes-Selva, Juan M. García-Gómez, Julia Gawronska, Maria Moudatsou, Ioanna Tabaki, Katerina Belogianni, Pania Karnaki, Miguel Rico Varadé, Rosa Gómez Trenado, Jaime Barrio-Cortes, Lee Smith, Alejandro Gil-Salmeron, Igor Grabovac

**Affiliations:** 1 Department of Social and Preventive Medicine, Centre for Public Health, Medical University of Vienna, Vienna, Austria; 2 Polibienestar Research Institute, University of Valencia, Valencia, Spain; 3 Biomedical Data Science Lab – ITACA Institute, Universitat Politecnica de Valencia, Valencia, Spain; 4 Centre for Health, Performance and Wellbeing, Anglia Ruskin University, Cambridge, UK; 5 PRAKSIS-Programs of Development, Social Support and Medical Cooperation-Non-Profitable Association, Athens, Greece; 6 Civil Law Non-Profit Organisation of Preventive Environmental and Occupational Medicine PROLEPSIS, Athens, Greece; 7 Department of Nutritional Sciences, Faculty of Life Sciences and Medicine, King’s College London, London, UK; 8 General Directorate of Social Services, Council of Family, Youth and Social Policy, Community of Madrid, Madrid, Spain; 9 Madrid Health Service SERMAS, Madrid, Spain; 10 Foundation for Research and Biosanitary Innovation in Primary Care, Madrid, Spain; 11 HM Faculty of Health Sciences, University Camilo José Cela, Madrid, Spain; 12 International Foundation for Integrated Care, Oxford, UK; 13 Comprehensive Cancer Center, Medical University of Vienna and Vienna General Hospital, Vienna, Austria

**Keywords:** Homelessness, Cancer prevention, Health navigation, Implementation research

## Abstract

**Background:**

People experiencing homelessness (PEH) face significant barriers to accessing cancer prevention services. The CANCERLESS project co-developed and implemented the Health Navigator Model (HNM) – a person-centred approach using trained health navigators to provide tailored support, promote cancer awareness, and facilitate access to preventive services – in Austria, Greece, Spain, and the UK. This study examined the HNM’s impact on healthcare empowerment, health-related quality of life (HRQoL), and self-rated health among PEH.

**Methods:**

This non-randomised, single-arm study included 652 PEH from 16 pilot sites in four European countries between June 2022 and December 2023. Healthcare empowerment, HRQoL, and self-rated health were assessed using the healthcare empowerment questionnaire and the EQ-5D-5L (including the EQ Visual Analogue Scale) when participants entered and completed the intervention. A per-protocol analysis with repeated measures ANOVA revealed changes over time and between countries.

**Results:**

Participants were predominantly male (64.1%) and middle-aged (mean 47.4, SD 13.8), with secondary education (58.0%), and varied legal status and experiences of homelessness. Despite a drop-out rate of 57.5%, significant improvements in overall healthcare empowerment (F = 44.325, *p*<.001, η²=0.026), HRQoL (F = 4.421, *p* = .036, η²=0.002), and self-rated health (F = 16.945, *p*<.001, η²=0.011) were observed, particularly in Austria and the UK.

**Conclusions:**

The HNM holds promise for improving healthcare empowerment and HRQoL among PEH, but context-specific factors and a considerable drop-out pose challenges.

**Trial registration:**

This study was registered retrospectively on June 6, 2022, in the registry of ClinicalTrials.gov under the number NCT05406687.

**Supplementary Information:**

The online version contains supplementary material available at https://doi.org/10.1186/s12955-026-02588-8.

## Introduction

Cancer remains a leading cause of morbidity and mortality worldwide [1], disproportionately impacting marginalised populations [2], with people experiencing homelessness (PEH) being particularly affected [3–6]. This disparity stems from a complex interplay of factors, including housing instability, high behavioural risk rates (such as smoking and substance use), financial constraints, and a lack of health insurance [7, 8]. Engendered by the experience of homelessness per se, deep-rooted mistrust in the healthcare system as well as a diminished sense of agency and control over one’s health often lead to fatalism or the marginalisation of preventive care in the face of immediate survival needs [8, 9].

Addressing this critical issue, the EU-funded CANCERLESS project focused on co-designing, piloting, and evaluating the Health Navigator Model (HNM) – a person-centred approach tailored to the unique cancer prevention-related needs of PEH in Austria, Greece, Spain, and the UK [10]. The HNM synthesises the Patient Navigation Model, which provides individualised assistance to overcome systemic barriers [11], and the Patient Empowerment Model, which fosters autonomy, self-management, and health advocacy [12]. Co-adapted through cross-national focus group discussions [13], the HNM utilises trained health and social care professionals or peers with lived experience to act as “health navigators”. These navigators actively assist users in identifying personal health needs, delivering tailored health literacy education, and coordinating direct access to primary and secondary cancer prevention services. By shifting individuals from passive recipients to active participants, the model seeks to systematically enhance both their healthcare empowerment and health-related quality of life (HRQoL).

In the context of cancer prevention, healthcare empowerment involves equipping individuals with the knowledge and skills to make informed decisions regarding screenings, risk-factor modification, and proactive communication with healthcare providers [14–17]. For underserved and highly marginalised populations such as PEH, fostering this baseline agency is critical to successfully navigating complex and often fragmented preventive care systems [18]. Similarly, HRQoL – encompassing physical, psychological, and social well-being [19] – is deeply undermined by the daily instabilities and chronic conditions associated with homelessness [20–22]. Thus, improving direct access to cancer preventive care not only holds the potential to mitigate cancer morbidity and mortality among PEH but can also enhance their healthcare empowerment and overall HRQoL by systematically addressing health needs that are frequently neglected in standard care settings.

This study aimed to assess the degree to which the HNM piloted within the CANCERLESS project impacted outcomes on healthcare empowerment and HRQoL among PEH receiving the intervention in Austria, Greece, Spain, and the UK. These four European countries present vastly distinct healthcare delivery frameworks that influence preventive health access for marginalised groups [9]. Spain and the UK operate tax-financed universal health coverage systems with established national cancer screening guidelines [23, 24], yet structural navigation and administrative hurdles frequently block PEH from utilising them. Austria has a social insurance scheme providing near-universal coverage, with deep systemic access gaps persisting for individuals lacking formal documentation or registration [25]. Meanwhile, Greece relies on a service coverage system where high out-of-pocket dynamics and fragmented prevention networks often force immediate survival needs to superimpose onto cancer screening priorities [26]. By examining the HNM across these diverse national frameworks, this study sought to evaluate macro-level country variations in the model’s performance to better inform future cross-national adaptations. This article was structured in accordance with the Transparent Reporting of Evaluations with Nonrandomized Designs (TREND) checklist developed to guide the reporting of behavioural and public health intervention evaluations [27].

## Methods

### Study design and participants

This study employed a longitudinal, non-randomised, single-arm design based on ethical considerations concerning the lack of directly comparable interventions while withholding a potentially beneficial service (the HNM intervention) from a control group of PEH, thereby potentially exacerbating health inequities by denying care [28, 29]. Moreover, alternative designs, such as randomised controlled trials, were deemed logistically unfeasible due to the high risk of cross-group contamination in shared community resource settings such as night shelters.

The participants in this study consisted of people experiencing any type of homelessness who were 18 years or older, had no cancer diagnosis, and had sufficient command of a language spoken at their respective pilot site. In this context, homelessness was classified as defined by the European Typology of Homelessness and Housing Exclusion (ETHOS), which serves as a comprehensive framework within professional and academic fields working with communities of PEH [30]. ETHOS differentiates homelessness into four conceptual categories, namely rooflessness, houselessness, living in insecure accommodation, and living in inadequate accommodation, and divides them into further operational categories and associated living situations. Individuals were excluded if they had a cognitive disability, were unable to give informed consent, or were living with or beyond cancer. Participants were recruited by health navigators directly at the pilot sites and through referrals from services and programmes with which cooperations existed. Recruitment occurred at the intervention sites, including outpatient clinics, day-care centres, shelters, support centres, and other community-based organisations. Health navigators collected data at the sites where the intervention was delivered, at recruitment and upon individual completion.

Before recruitment, a target sample size of 328 participants was determined. This number was computed based on detecting a small-to-medium effect size (f = 0.12) using repeated measures ANOVA investigating within-between interaction, assuming a two-tailed alpha level of 0.05, a power of 0.8, a correlation of 0.5 among repeated measures, and a drop-out rate of 40%. The unit of assignment was the individual participant. As there was only one study condition (the intervention group), random assignment was not employed, meaning participants, health navigators, and researchers were not blinded to group allocation, and group matching was not indicated. The individual participant served as the smallest unit of analysis for assessing intervention effects. Thus, the unit of analysis was the same as the unit of assignment, which allowed for a direct assessment of the intervention’s impact on individual participants.

### Health Navigator Model intervention

As a foundational part of the CANCERLESS project, the HNM intervention was implemented in 16 pilot sites (Table 1). Specific facilities and clinics were chosen based on several standardised selection criteria. These required that institutions demonstrated: experience working with PEH, prior involvement in research or projects addressing issues related to homelessness, supporting people from various socio-economic contexts, and the institutions’ availability and willingness to pilot implementation. The intervention aimed to empower PEH through various primary and secondary cancer prevention activities tailored to address individual needs that emerged during the implementation period. A total of 20 specially trained health navigators – who either had a professional health or social care background or were peer workers within participating organisations – were appointed at each pilot site, distributed across the countries as follows: Austria (*n* = 4), Spain (*n* = 4), Greece (*n* = 8), and the UK (*n* = 4). As part of the intervention, their training focused on increasing their knowledge of homelessness and cancer-related risk factors, enhancing communication skills, familiarising them with local health and social care systems, and developing competence in building trusting relationships with service facilitators and PEH users.

**Table 1:** Pilot sites for the Health Navigator Model implementation

Country	City	Pilot sites
Austria	Vienna	One outpatient clinic
		Two day-care centres
Greece	Athens	Two day-care centres
		One community centre
	Thessaloniki	One polyclinic
Spain	Madrid	Four shelters
		One street social service centre
UK	Cambridge	Two support centres
	King’s Lynn	One “move-through” hostel
	Norwich	One “move-through” hostel

Primary prevention activities conducted during the intervention included one-on-one appointments between health navigators and users to gather information and identify individual health needs. Navigators also guided users toward cancer prevention educational resources, such as health promotion and cancer awareness workshops, and facilitated access to vaccination programs and health-promoting facilities like gyms. Secondary prevention activities centred on navigator-guided referrals and arranging cancer screening appointments and other necessary medical support. Health navigators also actively facilitated access to these appointments by providing accompaniment and assisting with issues related to lacking or insufficient health insurance. Specifically, for participants lacking valid insurance or formal documentation, navigators provided individualised legal and administrative advocacy. This included filling out alternative funding applications, leveraging existing institutional partnerships with humanitarian and NGO-run outpatient clinics, and guiding users through complex bureaucratic processes to obtain emergency or temporary social health coverage. Furthermore, personalised support involved tailoring the frequency of check-ins to the participant’s housing stability and providing practical resources, such as public transport tickets and translation assistance, to ensure attendance at medical appointments, including cancer screenings. The unit of intervention delivery was primarily individual, focusing on the specific needs of each participant. Health navigators were the intervention deliverers, ensuring consistent and personalised support throughout the process, and collecting participants’ data. The intervention was delivered directly in the pilot site settings.

The intervention was designed to be delivered over a period of approximately 19 months, aligning with the overall study duration, which spanned from June 2022 to December 2023. Participants entered the study after recruitment, which involved health navigators approaching eligible PEH and informing them about the CANCERLESS project and the HNM. Those who expressed interest and provided informed consent completed a baseline questionnaire before receiving any intervention. During the intervention period, PEH engaged in the cancer prevention activities described above. The frequency and duration of interactions with the health navigators and engagement with resources were tailored to individual needs and preferences but were intended to last throughout the intervention period without extending beyond it. The intervention period culminated in a follow-up time point, where participants completed follow-up questionnaires, marking the conclusion of their participation in the study. No specific incentives were utilised to increase compliance or adherence, relying instead on the supportive and empowering nature of the health navigator relationship and the inherent value of the services provided. Nonetheless, all costs potentially associated with the intervention were covered for participants.

To monitor implementation fidelity and ensure data quality, a standardised, multi-tiered monitoring system was implemented throughout the study period. On a monthly basis, local researchers and health navigators at each pilot site completed a dedicated online form tracking feasibility metrics, including the number of individuals approached, the number of completed questionnaires at each evaluation timepoint, specific prevention activities facilitated, and structural barriers or facilitators encountered by health navigators. In parallel, monthly virtual meetings were held with all navigators and researchers from each local team to assess study progress, address data collection issues, and harmonise intervention delivery. Following each meeting, formalised minutes were disseminated across the teams to document agreed-upon actions. All collected feasibility metrics were continuously cross-checked with the raw study data by local researchers. Detailed information on the implementation fidelity monitoring has been reported elsewhere [31].

### Outcomes

All outcomes, except satisfaction with the received service, were measured at baseline and end of the HNM intervention, with a mean length of follow-up of 157.8 days (SD = 132.0; min = 28; max = 547). Primary outcomes focused on healthcare empowerment and HRQoL. Healthcare empowerment was assessed using the Healthcare Empowerment Questionnaire (HCEQ), a validated instrument to measure an individual’s perceived ability and motivation to manage personal healthcare [14]. Each item in the HCEQ is assessed using two sequential questions. First, participants are asked, “Did you feel that...” followed by a specific statement related to healthcare interaction, such as “...you asked for explanations.” The second question for each item assesses motivation by asking, “How important is it that...” followed by the same statement. Both response scales use a four-point Likert scale. The final score for each item is calculated by multiplying the response values from the two questions, creating a product that ranges from 1 to 16. The highest possible score of 16 indicates that the individual both felt a high degree of control and considered that aspect of control to be extremely important. Conversely, a score of 1 represents the lowest level of empowerment, indicating that the individual felt little to no control and did not consider that aspect of control important. The overall HCEQ score is derived by calculating the mean of product scores for all items.

HRQoL was measured using the EQ-5D-5L questionnaire, a widely recognised and standardised instrument with established psychometric properties that assesses health status across five dimensions: mobility, self-care, usual activities, pain/discomfort, and anxiety/depression, each with five levels of severity [32]. Using established country-specific EQ-5D-5L value sets [33–35], these responses are converted into an index score representing individuals’ overall health within their regional context. An index value of 1 signifies the best possible health state, while an index value below or equal to 0 signifies the worst possible health state (equivalent to death), allowing for a quantitative and standardised evaluation of participants’ HRQoL. In addition to the index value, the EQ-5D-5L comes with a visual analogue scale (EQ VAS) that assesses individuals’ self-rated health on a continuous scale from 0 to 100.

Secondary outcomes included additional factors potentially influenced by the intervention. Health literacy was evaluated using three validated health literacy screening questions briefly assessing an individual’s ability to understand written medical information, their confidence in completing medical forms independently, and their need for assistance in reading hospital materials, higher scores on a Likert scale from 0 to 4 indicating poorer degrees of health literacy [36]. General quality of life was measured using two generic questions derived from the WHOQOL-BREF, a comprehensive instrument assessing quality of life across various domains on a Likert scale ranging from 1 to 5 [37]. Satisfaction with healthcare in general and services offered by health navigators was measured using ad-hoc questions post-intervention. These questions assessed participant satisfaction with support received, helpfulness of organised care, and overall satisfaction with the service on a Likert scale from 0 to 4. Finally, interpersonal communication with health and social care providers was assessed using the person-centred coordinated care experience questionnaire (P3CEQ), an instrument designed to capture patients’ perspectives on the quality of communication with their healthcare providers on a Likert scale ranging from 0 to 3 [38]. Higher scores in general quality of life, satisfaction, and interpersonal communication indicate better outcomes.

Health navigators collected data from participating PEH, which was then gathered into a dedicated online platform created and managed specifically for this study [39]. Participants’ sociodemographic information and responses were collected through paper questionnaires, with data subsequently entered into the online platform, or directly inputted via electronic devices. To ensure consistency across the diverse linguistic environments, all collected local-language data were populated directly into this standardised platform, which automatically harmonised translated instruments into a uniform dataset.

### Statistical methods

Descriptive statistics were employed to summarise participant characteristics at baseline and outcomes at both baseline (pre-intervention) and follow-up (post-intervention). To assess the impact of the HNM intervention on the outcomes in question, repeated measures ANOVA was performed. This method was chosen to account for the paired nature of the data and allowed to determine whether there were statistically significant changes in outcomes over time. While Shapiro-Wilk tests revealed non-normal distribution of the relevant outcome data, repeated measures ANOVA was deemed appropriate due to its robustness against violations of normality [40]. The statistical models included time (baseline vs. follow-up) as a within-subject factor and pilot site locations as between-subject factors to explore potential differences in outcome changes across the four countries. Effect sizes were calculated and reported using eta squared (η²) for the omnibus test and Cohen’s d for post-hoc tests. Due to the lack of a control group and the non-randomised nature of the study, the primary analysis focused on within-group changes from baseline to follow-up. The significance level for all statistical tests was set at *p* < .05.

While repeated measures ANOVA was conducted following a per-protocol approach and only included those who completed the follow-up assessments, the descriptive analysis encompassed data from all participants. Therefore, the results of both descriptive and inferential analyses were compared and interpreted in conjunction to provide a comprehensive understanding of the intervention’s effects. Changes in mean scores observed in the descriptive analysis were evaluated alongside the statistical significance determined by the repeated measures ANOVA, enabling the assessment of the practical importance of changes observed in relevant outcomes.

To investigate potential attrition bias, a descriptive approach was used to compare the characteristics of participants who completed the follow-up assessment with those who dropped out. Chi-square tests were used for categorical variables and independent t-tests for continuous variables to assess whether there were significant differences in baseline characteristics between those who completed the study and those who were lost to follow-up.

Data analysis was conducted using JASP version 0.19.3 [41].

## Results

**Fig. 1:**
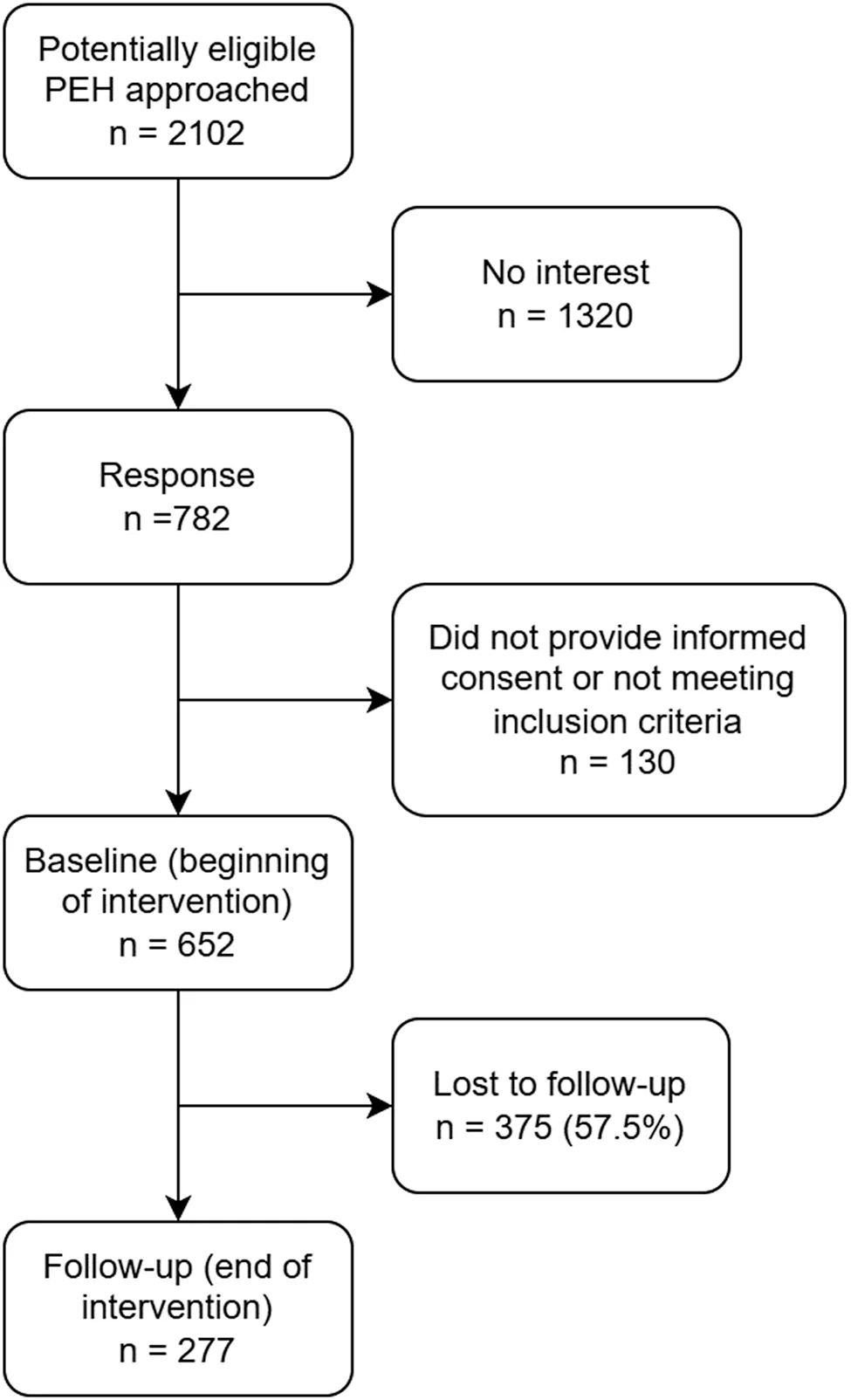
Participant flow chart. PEH: People experiencing homelessness

Recruitment started in June 2022 and ended in November 2023. As illustrated in Fig. 1, the participant flow indicates that out of the 2,102 PEH approached, 782 responded to the invitation, and 652 consented to participate. Given the study’s single-arm design, all enrolled participants received the HNM intervention. Out of the total number of participants, 277 (42.5%) completed the follow-up, with the last assessment taking place in December 2023. The primary reasons for dropout included loss of contact (*n* = 84), loss of interest (*n* = 20), and competing priorities (*n* = 11). Details of participants’ baseline sociodemographics are presented in Table 2.

**Table 2:** Baseline demographics of study participants

	Total (*n* = 652)	Austria (*n* = 142)	Greece (*n* = 158)	Spain (*n* = 197)	United Kingdom (*n* = 155)
**Age, mean (SD)**	47.4 (13.8)	45.3 (12.8)	51.6 (13.5)	49.0 (14.8)	42.9 (12.0)
**Gender**,** n (%)**					
Male	418 (64.1)	60 (42.3)	121 (76.6)	110 (55.8)	127 (81.9)
Female	230 (35.3)	82 (57.7)	34 (21.5)	87 (44.2)	27 (17.4)
Non-binary	1 (0.2)	0 (0.0)	0 (0.0)	0 (0.0)	1 (0.6)
Other	2 (0.3)	0 (0.0)	2 (1.3)	0 (0.0)	0 (0.0)
**Level of education (ISCED 2011)**,** n (%)**					
Early childhood education	29 (4.4)	10 (7.0)	9 (5.7)	8 (4.1)	2 (1.3)
Primary education	95 (14.6)	12 (8.5)	43 (27.2)	30 (15.2)	10 (6.5)
Lower secondary education	182 (27.9)	73 (51.4)	47 (29.7)	47 (23.9)	15 (9.7)
Upper secondary education	196 (30.1)	27 (19.0)	26 (16.5)	57 (28.9)	86 (55.5)
Post-secondary non-tertiary education	45 (6.9)	1 (0.7)	10 (6.3)	12 (6.1)	22 (14.2)
Short-cycle tertiary education	19 (2.9)	5 (3.5)	6 (3.8)	6 (3.0)	2 (1.3)
Bachelor’s or equivalent	56 (8.6)	4 (2.8)	16 (10.1)	20 (10.2)	16 (10.3)
Master’s or equivalent	5 (0.8)	1 (0.7)	1 (0.6)	1 (0.5)	2 (1.3)
Doctorate or equivalent	0 (0.0)	0 (0.0)	0 (0.0)	0 (0.0)	0 (0.0)
**Legal status**,** n (%)**					
National citizen	405 (62.1)	16 (11.3)	142 (89.9)	118 (59.9)	129 (83.2)
Asylum seeker	108 (16.6)	15 (10.6)	10 (6.3)	61 (31.0)	22 (14.2)
Refugee	11 (1.7)	6 (4.2)	3 (1.9)	0 (0.0)	2 (1.3)
No legal documents	120 (18.4)	104 (73.2)	3 (1.9)	12 (6.1)	1 (0.6)
**Time experiencing homelessness**,** n (%)**					
Up to 3 months	86 (13.2)	22 (15.5)	9 (5.7)	11 (5.6)	44 (28.4)
3 months to 1 year	145 (22.2)	49 (34.5)	29 (18.4)	38 (19.3)	29 (18.7)
1 to 3 years	123 (18.9)	26 (18.3)	25 (15.8)	43 (21.8)	29 (18.7)
3 to 5 years	88 (13.5)	15 (10.6)	30 (19.0)	30 (15.2)	13 (8.4)
5 to 10 years	94 (14.4)	16 (11.3)	28 (17.7)	32 (16.2)	18 (11.6)
More than 10 years	112 (17.2)	13 (9.2)	37 (23.4)	40 (20.3)	22 (14.2)
**Previous homelessness**,** n (%)**	339 (52.0)	126 (88.7)	114 (72.2)	42 (21.3)	57 (36.8)
**ETHOS category**,** n (%)**					
People Living Rough	92 (14.1)	5 (3.5)	34 (21.5)	6 (3.0)	47 (30.3)
People in emergency accommodation	170 (26.1)	3 (2.1)	56 (35.4)	107 (54.3)	4 (2.6)
People in accommodation for PEH	138 (21.2)	3 (2.1)	14 (8.9)	53 (26.9)	68 (43.9)
People in Women’s Shelter	34 (5.2)	2 (1.4)	0 (0.0)	31 (15.7)	1 (0.6)
People in accommodation for immigrants	3 (0.5)	2 (1.4)	1 (0.6)	0 (0.0)	0 (0.0)
People due to be released from institutions	4 (0.6)	4 (2.8)	0 (0.0)	0 (0.0)	0 (0.0)
People receiving longer-term support (due to homelessness)	114 (17.5)	79 (55.6)	18 (11.4)	0 (0.0)	17 (11.0)
People living in insecure accommodation	49 (7.5)	39 (27.5)	1 (0.6)	0 (0.0)	9 (5.8)
People living under threat of eviction	1 (0.2)	0 (0.0)	1 (0.6)	0 (0.0)	0 (0.0)
People living under threat of violence	1 (0.2)	0 (0.0)	1 (0.6)	0 (0.0)	0 (0.0)
People living in temporary/non-conventional structures	38 (5.8)	0 (0.0)	30 (19.0)	0 (0.0)	8 (5.2)
People living in unfit housing	5 (0.8)	3 (2.1)	2 (1.3)	0 (0.0)	0 (0.0)
People living in extreme over-crowding	0 (0.0)	0 (0.0)	0 (0.0)	0 (0.0)	0 (0.0)
**Source of income**,** n (%)**					
None	224 (34.4)	77 (54.2)	19 (12.0)	115 (58.4)	13 (8.4)
Work	72 (11.0)	32 (22.5)	14 (8.9)	16 (8.1)	10 (6.5)
Social benefits	272 (41.7)	17 (12.0)	107 (67.7)	20 (10.2)	128 (82.6)
Others	84 (12.9)	16 (11.3)	18 (11.4)	46 (23.4)	4 (2.6)
**Amount of income**,** mean (SD)**	Not calculable due to mixed currencies	535.00 EUR (350.9)	300.70 EUR (191.5)	446.70 EUR (311.5)	418.96 GBP (418.96)
**Access to public health services**,** n (%)**	323 (49.5)	16 (11.3)	26 (16.5)	126 (64.0)	155 (100.0)

ETHOS: European Typology of Homelessness and Housing Exclusion. ISCED: International Standard Classification of Education. PEH: People experiencing homelessness

Participants demonstrated an overall improvement in healthcare empowerment from baseline to follow-up (Table 3). At baseline, participants in Spain had the highest mean HCEQ score, followed by those in the UK, Austria, and Greece. Participants in all countries except Greece exhibited an increase in scores at follow-up, those in Austria showing the most substantial improvement, and those in the UK also demonstrating a notable increase. Participants in Spain also showed an increase in their HCEQ score, while the score of the Greek sample decreased slightly. For HRQoL, as measured by the EQ-5D-5L index, a slight overall increase was observed from baseline to follow-up. At baseline, participants in Austria had the highest HRQoL index score, followed by those in Greece, Spain, and the UK. At follow-up, participants in Austria and the UK improved their scores, while those in Greece and Spain remained relatively stable. The EQ VAS scores, which reflect participants’ self-rated health, also improved overall from baseline to follow-up. At baseline, participants in Greece had the highest VAS score, followed by those in Austria, Spain, and the UK. The Austrian sample demonstrated the most substantial improvement at follow-up, and that of the UK also showed an increase. While participants’ scores decreased slightly in Spain, they did not change notably in the Greek sample.

Participants reported a slight overall improvement in their general quality of life, with those in Austria showing a slight improvement at follow-up, while it remained relatively stable or slightly decreased in PEH at Greek, Spanish, and UK pilot sites. Overall, satisfaction with health increased slightly, mainly due to a strong increase in participants in Austria. In Greece and Spain, participants’ scores remained relatively stable, while decreased in scores of those in the UK. Health literacy scores remained relatively stable overall and across all countries’ samples, indicating consistently low health literacy among participants. Satisfaction with healthcare and HNM services (measured at follow-up only) was highest in participants in Austria, followed by those in the UK, Greece, and Spain. Finally, P3CEQ scores improved overall, with PEH in Austria and the UK showing the most improvement at follow-up, scores of those in Spain having increased slightly and of those in Greece having experienced a decrease. Details on this descriptive analysis can be found in Table 3.

**Table 3:** Primary and secondary outcomes of study participants at baseline and follow-up

	Total		Austria		Greece		Spain		United Kingdom	
**Primary outcomes, mean (SD)**										
	**Baseline (** ***n*** ** = 652)**	**Follow-up (** ***n*** ** = 277)**	**Baseline (** ***n*** ** = 142)**	**Follow-up (** ***n*** ** = 66)**	**Baseline (** ***n*** ** = 158)**	**Follow-up (** ***n*** ** = 111)**	**Baseline (** ***n*** ** = 197)**	**Follow-up (** ***n*** ** = 35)**	**Baseline (** ***n*** ** = 155)**	**Follow-up (** ***n*** ** = 65)**
Healthcare empowerment (HCEQ)	6.69 (3.22)	7.53 (3.57)	6.93 (2.14)	9.09 (2.11)	5.25 (2.24)	5.19 (1.88)	6.49 (2.44)	8.42 (1.99)	8.10 (4.65)	9.37 (5.09)
HRQoL (EQ-5D-5 L index)	0.79 (0.25)	0.81 (0.22)	0.87 (0.18)	0.92 (0.13)	0.82 (0.19)	0.79 (0.22)	0.78 (0.25)	0.77 (0.21)	0.67 (0.30)	0.74 (0.25)
Self-rated health (EQ VAS)	60.85 (22.82)	66.92 (21.24)	64.68 (17.04)	76.78 (15.56)	69.99 (22.39)	70.23 (19.45)	56.43 (24.09)	54.41 (26.96)	52.19 (22.59)	58.09 (19.56)
**Secondary outcomes**,** mean (SD)**										
	**Baseline**	**Follow-up**	**Baseline**	**Follow-up**	**Baseline**	**Follow-up**	**Baseline**	**Follow-up**	**Baseline**	**Follow-up**
How would you rate your quality of life? (WHOQOL-BREF)	2.74 (1.09)	2.75 (1.07)	3.03 (0.88)	3.33 (0.73)	2.34 (1.02)	2.42 (1.00)	3.00 (1.14)	3.11 (0.96)	2.57 (1.10)	2.52 (1.24)
How satisfied are you with your health? (WHOQOL-BREF)	2.98 (1.18)	3.10 (1.17)	2.85 (1.00)	3.38 (0.80)	3.30 (1.13)	3.32 (1.14)	3.11 (1.24)	3.06 (1.24)	2.60 (1.18)	2.46 (1.29)
Health literacy (health literacy screening)	1.32 (1.24)	1.47 (1.16)	1.96 (1.39)	1.94 (1.33)	1.48 (0.99)	1.53 (0.97)	1.00 (1.21)	1.03 (1.12)	0.98 (1.12)	1.11 (1.13)
Satisfaction with healthcare and HNM services	-	2.89 (0.42)	-	3.23 (0.34)	-	2.78 (0.31)	-	2.67 (0.47)	-	2.82 (0.43)
Interpersonal communication (P3CEQ)	2.42 (0.42)	2.65 (0.48)	2.73 (0.15)	2.98 (0.15)	2.22 (0.42)	2.08 (0.61)	2.20 (0.35)	2.56 (0.37)	2.15 (0.47)	2.44 (0.53)

HNM: Health navigator model. Note: Comprehensive post-hoc pairwise comparisons and significant within-subject changes over time and between-country differences are detailed in the main text and Supplemental Table S1

The repeated measures ANOVA revealed significant within-subject effects (changes over time) and between-subject effects (differences between pilot countries) for several outcomes. For healthcare empowerment, a significant main effect of time was found (F = 44.325, *p*<.001, η²=0.026), indicating that healthcare empowerment scores changed significantly from baseline to follow-up across all countries’ samples. A significant main effect of pilot country was also observed (F = 29.260, *p*<.001, η²=0.209), suggesting differences in healthcare empowerment scores between the four countries. Furthermore, a significant interaction effect between time and pilot country was found (F = 10.507, *p*<.001, η²=0.018), indicating that the change in healthcare empowerment scores over time differed across the four countries.

Regarding HRQoL, as measured by the EQ-5D-5L index, a significant main effect of time was observed (F = 4.421, *p* = .036, η²=0.002), indicating a significant change in HRQoL index scores from baseline to follow-up. A significant main effect of pilot country was found (F = 10.538, *p*<.001, η²=0.089), suggesting differences in HRQoL index scores between countries. A significant interaction effect between time and pilot country was also observed (F = 4.193, *p* = .006, η²=0.006), indicating that the change in HRQoL index scores over time differed across countries. For self-rated health, measured by the EQ VAS, a significant main effect of time was found (F = 16.945, *p*<.001, η²=0.011), indicating a significant change in VAS scores from baseline to follow-up. A significant main effect of pilot country was observed (F = 18.785, *p*<.001, η²=0.139), suggesting differences in VAS scores between countries. A significant interaction effect between time and pilot country was found (F = 5.220, *p* = .002, η²=0.010), indicating that the change in VAS scores over time differed across countries.

General quality of life and satisfaction with health showed significant improvements over time, with significant differences between countries and significant interaction effects. Health literacy did not change significantly overall, but differences were found between countries, and the interaction effect suggested that changes over time in health literacy differed across countries. Finally, interpersonal communication improved significantly over time, with significant differences between countries and a significant interaction effect, indicating varying changes across countries.

The post hoc analysis, using pairwise comparisons, investigated the significant effects found in the repeated measures ANOVA for the primary outcomes (see Supplemental Table S1). For healthcare empowerment, at baseline, participants in Austria, the UK, and Spain had significantly higher scores than those in Greece. At follow-up, PEH in Austria had higher scores than those in Greece and Spain, and participants in the UK had higher scores than those in Greece. Within-country comparisons showed significant increases in HCEQ scores from baseline to follow-up for participants in Austrian, Spanish, and UK pilot sites. Regarding the EQ-5D-5L index for HRQoL, at baseline, participants in Austria had significantly higher scores than those in the UK. At follow-up, PEH in Austria still had higher scores than PEH in the UK. Within-country comparisons showed significant increases in HRQoL index scores for participants in Austria and the UK from baseline to follow-up. For the EQ VAS, at baseline, participants in Austria had lower scores than those in Greece but higher scores than those in the UK. Participants in Greece and Spain also had higher scores than those in the UK. At follow-up, PEH in Austria had higher scores than those in Spain and the UK. Within-country comparisons showed significant increases in VAS scores for participants in Austria and the UK from baseline to follow-up.

**Fig. 2:**
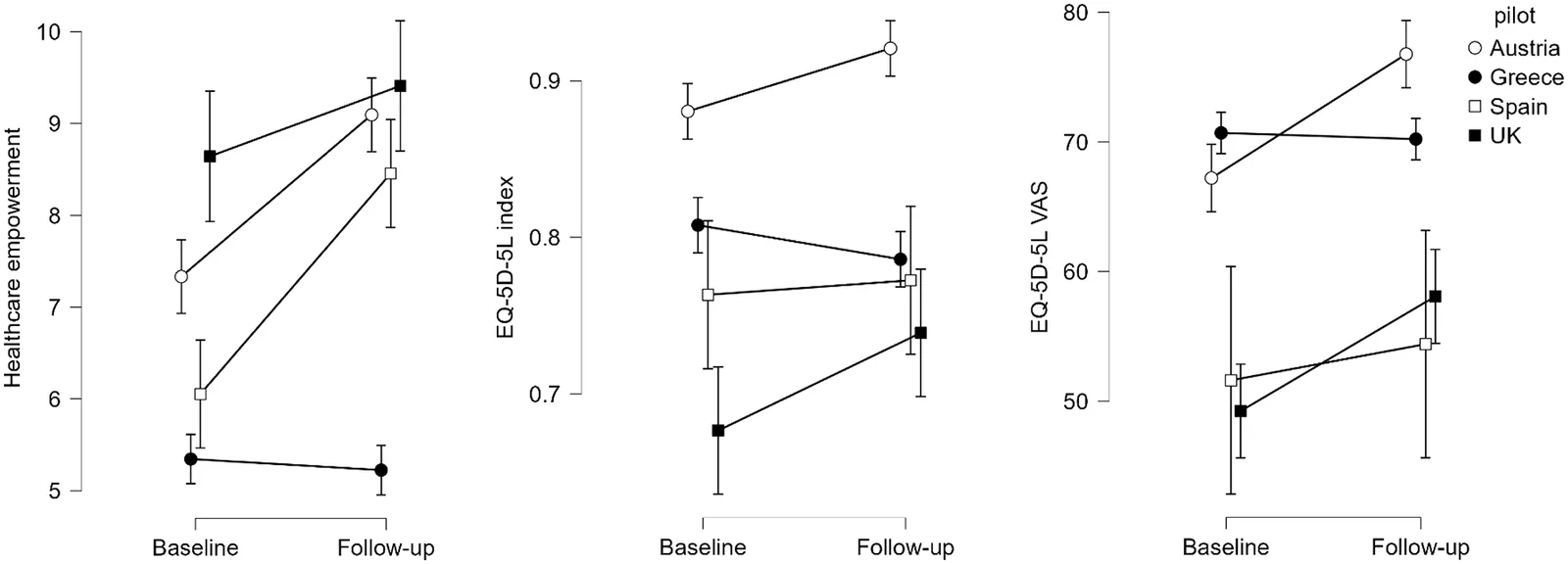
Changes in primary outcome mean scores over time across pilot countries showing 95% confidence intervals

Changes in outcome means over time for each country are illustrated in Fig. 2 (with comprehensive distributions displayed in Supplemental Figure S1).

To assess potential attrition bias, baseline characteristics of participants who completed the follow-up assessment (*n* = 277) and those who dropped out (*n* = 375) were compared. No significant differences were found between the two groups in terms of gender, education, legal status, or income. However, participants who completed the study were significantly older than those who dropped out (mean 49.37 (SD 13.27) vs. mean 45.89 (SD 14.02), t = 3.204, 95%CI 1.347 to 5.613). The distribution across ETHOS categories also differed significantly between the two groups (χ^2^ = 48.922, *p*<.001), with a higher proportion of completers receiving longer-term support and a higher proportion of dropped-out participants being in emergency accommodation or women’s shelters. Finally, a significantly larger proportion of participants who dropped out had no access to public health services compared to those who completed the study (χ^2^ = 12.865, *p*<.001).

## Discussion

The findings of this study provide insights into the effectiveness of the HNM intervention concerning its impact on healthcare empowerment and HRQoL in PEH across Austria, Greece, Spain, and the UK. The HNM demonstrated a positive and significant impact on both healthcare empowerment and HRQoL. These findings align with previous research highlighting the positive impact of patient navigation programmes on (preventive) health and care outcomes among marginalised communities [42–44]. However, the results also underscore the importance of context, as the intervention’s effectiveness varied considerably across the four pilot countries.

The significant improvement in healthcare empowerment observed across all countries suggests that the HNM successfully equipped PEH with the knowledge, skills, and confidence to navigate the healthcare system and engage in shared decision-making. This is a crucial finding, given that empowerment is a key determinant of health behaviour change and adherence to medical recommendations [15, 18]. Nevertheless, several factors might explain the lack of significant improvement in healthcare empowerment in Greece. Systemic barriers within the healthcare system, cultural factors, or variations in sample characteristics or implementing the intervention may have presented challenges, as PEH could have prioritised more immediate (health) needs over cancer prevention actions. Further qualitative research exploring the perspectives of PEH and health navigators in Greece could shed light on these factors.

Observed improvements in HRQoL may be a result of enhancing preventive care access and positively impacting participants’ overall well-being. These findings are consistent with a previous study demonstrating the link between empowerment and improved HRQoL in PEH [20]. The improvements in self-rated health further support this conclusion. Remarkably, the variation in HRQoL outcomes across countries highlights the complex interplay of factors that influence well-being among PEH. While the HNM likely contributed to improvements in the Austrian and UK samples, less pronounced changes were observed in participants in Greece and Spain, potentially due to factors such as differences in social support systems, housing stability, and access to mental health services. However, wide confidence intervals observed in HRQoL, particularly in the Spanish sample, limit conclusions about the magnitude of this change, thus necessitating cautious interpretation of these findings.

The pronounced cross-national variations in healthcare empowerment and HRQoL outcomes reflect how the HNM dynamically interacts with distinct macro-level healthcare delivery frameworks. Spain and the United Kingdom both operate tax-financed universal health coverage frameworks, where foundational access to care is theoretically guaranteed [23, 24], yet navigating complex administrative and structural pathways remains a considerable obstacle for PEH [7, 9]. In these settings, the health navigator functioned as an effective systemic bridge to tackle these navigation hurdles, which likely explains the significant within-country improvements observed in healthcare empowerment across both the Spanish and British cohorts. However, while the UK sample also saw notable gains in HRQoL, the Spanish cohort’s HRQoL and self-rated health metrics remained relatively stable. This divergence, coupled with the wide confidence intervals observed in the Spanish sample, suggests that overcoming structural navigation barriers alone may be insufficient to shift overall well-being if deeper, competing systemic vulnerabilities – such as localised disparities in social support frameworks, acute housing instability, or fragmented access to mental health services – remain unaddressed [45, 46]. A similar bridging mechanism may explain the substantial success observed in Austria’s social insurance scheme. While this model typically excludes individuals lacking formal registration or documentation [25], the co-location of navigators within a well-developed, robust community network allowed the intervention to bypass these systemic insurance gaps through individualised administrative advocacy and institutional partnerships.

Conversely, the lack of significant improvement across primary metrics in Greece highlights the limitations of an isolated navigation intervention when deployed within a fundamentally fragmented system characterised by high out-of-pocket costs [26]. In such environments, systemic resource deficits and financial barriers may render a navigation model insufficient, as these structural barriers continuously force immediate survival and material needs to overshadow the focus on longer-term cancer prevention [47]. Specifically, within the Greek healthcare system, a severe deficiency in integrated public primary care networks forces individuals to interface directly with tertiary hospital settings where rigid bureaucratic and administrative hurdles are often in place [48]. For PEH, navigating these environments without a formal, continuous support track often harbours systemic alienation rather than agency. Moreover, because of the Greek system’s out-of-pocket dynamics, PEH may anticipate hidden costs even for theoretically subsidised preventive services. This financial anxiety reinforces a psychological barrier: when immediate survival needs are compounded by the threat of unaffordable medical care, an individual’s perceived capability to actively manage their healthcare – the core of empowerment – remains profoundly restricted [49]. Consequently, an isolated health navigation model cannot easily generate baseline agency when operating inside a structurally fragmented system that lacks robust, accessible safety nets.

The stable levels of health literacy across all countries highlight a persistent challenge in communicating health information effectively to marginalised populations. This is particularly relevant for PEH, who often face multiple barriers to accessing and understanding health information [7]. Future iterations of the HNM could incorporate more intensive health literacy interventions tailored to the specific needs of PEH, for example, through peer support models where individuals with lived experience assist in delivering information and support [50]. Furthermore, partnering with adult literacy programmes or community organisations could provide additional support in addressing the underlying causes of low health literacy.

The improvements in general quality of life and satisfaction with health are promising and suggest that the HNM positively impacted participants’ overall well-being. Notably, higher satisfaction with healthcare and the provided HNM services were reported in Austria. However, this circumstance might not necessarily reflect a more seamless integration of the HNM with the existing Austrian healthcare system, which often excludes people without health insurance, but may be an outcome of a more developed and robust community network of care established through health navigators and potentially facilitated by co-location within community organisations. Additionally, higher satisfaction scores may be a result of the HNM enhancing the provision of usual care by offering comprehensive services, and it is possible that the training and support provided to health navigators in Austria were particularly effective in fostering strong relationships with participants, leading to increased trust and satisfaction. Lastly, the organisational culture within the Austrian pilot sites might have been more aligned with the principles of person-centred care.

### Limitations

Several limitations should be considered when interpreting these findings. The longitudinal, non-randomised, single-arm design of the study significantly limits the ability to draw causal inferences. While the repeated measures ANOVA controlled for within-subject variability, the lack of a control group makes it difficult to attribute the observed changes solely to the HNM intervention. Furthermore, the flexibility of the intervention, while intended to be person-centred, means that not all PEH received the same level of cancer preventive activities (e.g., raising cancer awareness). Additionally, the study’s follow-up period might not have been long enough to capture the full impact of the HNM intervention, and variations in implementation fidelity, due to differences in local contexts, healthcare systems, and the skills, profiles, and experiences of individual health navigators, likely played a substantial role in the observed results. Participants were recruited from specific pilot sites and were not randomly selected from the broader population of PEH in each country, introducing potential selection bias. Those who agreed to participate might have been more motivated, had better health status to begin with, or had more positive experiences with healthcare services than those who did not participate. Consequently, the study sample might not be fully representative of all PEH in the four countries, limiting the generalisability of the findings. Another significant limitation is the relatively high dropout rate of 57.5%, which is common in studies involving marginalised populations [51]. Due to this high dropout rate, data imputation was not a viable strategy to address missing data. The analysis of attrition bias revealed that those who completed the study were older, tended to receive longer-term homelessness support, and had more access to public health services than those who dropped out. This suggests that attrition was not completely random and that the results may be less generalisable to younger PEH, those in emergency accommodation and women’s shelters, and those without access to public health services. Finally, although validated instruments were used, reliance on self-reported data for most outcomes introduces the potential for recall bias and the subjective nature of these self-reports should be considered. In particular, because the post-intervention satisfaction data, among other outcomes, were collected directly by the health navigators who delivered the intervention, a notable risk of social desirability bias is present. Participants experiencing homelessness may have over-reported their satisfaction with the service due to gratitude, the strong interpersonal rapport established with their assigned navigator, or concerns about losing access to intervention-related benefits if they provided negative feedback.

## Conclusion

The HNM intervention demonstrated promising results in improving healthcare empowerment and HRQoL among PEH in four European countries. The findings highlight the importance of person-centred, integrated care approaches that address this marginalised community’s unique needs and challenges related to cancer prevention. While further research is needed to confirm these findings and explore the long-term effects of the HNM, this study provides a robust foundation for implementing and scaling up similar interventions to improve the health and well-being of PEH in Europe and beyond. By empowering PEH to navigate the healthcare system and engage proactively in their care, the HNM has the potential to reduce disparities in cancer preventive care and promote health equity for this often-neglected population.

## Supplementary Information

Below is the link to the electronic supplementary material.

Supplementary Material 1

## Data Availability

The datasets analysed during the current study are not publicly available due to data sensitivity and are available from the corresponding author upon reasonable request.

## References

[1] Bray F, Laversanne M, Sung H, Ferlay J, Siegel RL, Soerjomataram I, Jemal A. Global cancer statistics 2022: GLOBOCAN estimates of incidence and mortality worldwide for 36 cancers in 185 countries. CA Cancer J Clin. 2024;74:229–63. 10.3322/caac.21834. 38572751

[2] Aldridge RW, Story A, Hwang SW, Nordentoft M, Luchenski SA, Hartwell G, et al. Morbidity and mortality in homeless individuals, prisoners, sex workers, and individuals with substance use disorders in high-income countries: a systematic review and meta-analysis. Lancet. 2018;391:241–50. 10.1016/S0140-6736(17)31869-X. PMC5803132 29137869

[3] Aldridge RW, Menezes D, Lewer D, Cornes M, Evans H, Blackburn RM, et al. Causes of death among homeless people: a population-based cross-sectional study of linked hospitalisation and mortality data in England. Wellcome Open Res. 2019;4:49. 10.12688/wellcomeopenres.15151.1. PMC6449792 30984881

[4] Holowatyj AN, Heath EI, Pappas LM, Ruterbusch JJ, Gorski DH, Triest JA, et al. The Epidemiology of Cancer Among Homeless Adults in Metropolitan Detroit. JNCI Cancer Spectr. 2019;3:pkz006. 10.1093/jncics/pkz006. PMC6433093 30944890

[5] Funk AM, Greene RN, Dill K, Valvassori P. The Impact of Homelessness on Mortality of Individuals Living in the United States: A Systematic Review of the Literature. J Health Care Poor Underserved. 2022;33:457–77. 10.1353/hpu.2022.0035. 35153234

[6] Roncarati JS, Baggett TP, O’Connell JJ, Hwang SW, Cook EF, Krieger N, Sorensen G. Mortality Among Unsheltered Homeless Adults in Boston, Massachusetts, 2000–2009. JAMA Intern Med. 2018;178:1242–8. 10.1001/jamainternmed.2018.2924. PMC6142967 30073282

[7] Carmichael C, Schiffler T, Smith L, Moudatsou M, Tabaki I, Doñate-Martínez A, et al. Barriers and facilitators to health care access for people experiencing homelessness in four European countries: an exploratory qualitative study. Int J Equity Health. 2023;22:206. 10.1186/s12939-023-02011-4. PMC10559410 37803317

[8] Jeleff M, Haider S, Schiffler T, Gil-Salmerón A, Yang L, Barreto Schuch F, Grabovac I. Cancer risk factors and access to cancer prevention services for people experiencing homelessness. Lancet Public Health. 2024;9:e128–46. 10.1016/S2468-2667(23)00298-0. 38307679

[9] Schiffler T, Carmichael C, Smith L, Doñate-Martínez A, Alhambra-Borrás T, Varadé MR, et al. Access to cancer preventive care and program considerations for people experiencing homelessness across four European countries: an exploratory qualitative study. EClinicalMedicine. 2023;62:102095. 10.1016/j.eclinm.2023.102095. PMC10393536 37533422

[10] CORDIS. Cancer prevention and early detection among the homeless population in Europe: Co-adapting and implementing the Health Navigator Model. 05.08.2024. https://cordis.europa.eu/project/id/965351. Accessed 28 Feb 2024.

[11] Freeman HP. The origin, evolution, and principles of patient navigation. Cancer Epidemiol Biomarkers Prev. 2012;21:1614–7. 10.1158/1055-9965.EPI-12-0982. 23045534

[12] World Health Organization. Health Promotion Glossary. 16.06.1998. https://iris.who.int/bitstream/handle/10665/64546/WHO_HPR_HEP_98.1.pdf?sequence=1. Accessed 12 Dec 2024.

[13] Gil-Salmerón A, Carmichael C, Fragner T, Moudatsou M, Tabaki I, Cortes JB, et al. Cancer Prevention and Screening for People Experiencing Homelessness: Co-Designing the Health Navigator Model. Int J Integr Care. 2025;25:11. 10.5334/ijic.9293. PMC12617427 41244881

[14] Gagnon M, Hibert R, Dubé M, Dubois M-F. Development and validation of an instrument measuring individual empowerment in relation to personal health care: the Health Care Empowerment Questionnaire (HCEQ). Am J Health Promot. 2006;20:429–35. 10.4278/0890-1171-20.6.429. 16871823

[15] Briss P, Rimer B, Reilley B, Coates RC, Lee NC, Mullen P, et al. Promoting informed decisions about cancer screening in communities and healthcare systems. Am J Prev Med. 2004;26:67–80. 10.1016/j.amepre.2003.09.012. 14700715

[16] Rathod S, Potdar J, Gupta A, Sethi N, Dande A. Empowering Women’s Health: Insights Into HPV Vaccination and the Prevention of Invasive Cervical Cancer. Cureus. 2023;15:e49523. 10.7759/cureus.49523. PMC10752828 38156129

[17] Russell CA, Régnier Denois V, Rouèche E, Simon V, Chauvin F. Empowerment Through Participation in a Cancer Prevention Exhibit: Evidence from the Hygée Lab Intervention. J Cancer Educ. 2021;36:519–31. 10.1007/s13187-019-01658-4. 31808052

[18] Rivera-Colón V, Ramos R, Davis JL, Escobar M, Inda NR, Paige L, et al. Empowering underserved populations through cancer prevention and early detection. J Community Health. 2013;38:1067–73. 10.1007/s10900-013-9715-8. 23807496

[19] Hays RD, Reeve BB. Measurement and modeling of health-related quality of life. International Encyclopedia of Public Health. Elsevier; 2008. pp. 241–52. 10.1016/B978-012373960-5.00336-1.

[20] Gadermann AM, Hubley AM, Russell LB, Palepu A. Subjective Health-Related Quality of Life in Homeless and Vulnerably Housed Individuals and Its Relationship with Self-Reported Physical and Mental Health Status. Soc Indic Res. 2014;116:341–52. 10.1007/s11205-013-0302-2.

[21] Lewer D, Aldridge RW, Menezes D, Sawyer C, Zaninotto P, Dedicoat M, et al. Health-related quality of life and prevalence of six chronic diseases in homeless and housed people: a cross-sectional study in London and Birmingham, England. BMJ Open. 2019;9:e025192. 10.1136/bmjopen-2018-025192. PMC6501971 31023754

[22] Spector AL, Quinn KG, McAuliffe TL, DiFranceisco W, Bendixen A, Dickson-Gomez J. Health-related quality of life and related factors among chronically homeless adults living in different permanent supportive housing models: a cross-sectional study. Qual Life Res. 2020;29:2051–61. 10.1007/s11136-020-02482-w. PMC7367711 32222931

[23] Bernal-Delgado E, Angulo-Pueyo E, Ridao-López M, Urbanos-Garrido RM, Oliva-Moreno J, García-Abiétar D, Hernández-Quevedo C. Spain: health system review. 2024. https://iris.who.int/server/api/core/bitstreams/484a7fc9-b55a-4994-aaa2-b9aa2cc77910/content. Accessed 6 Jul 2026. 40094303

[24] Anderson M, Pitchforth E, Edwards N, Alderwick H, McGuire A, Mossialos E. The United Kingdom: health system review. 2022. https://iris.who.int/server/api/core/bitstreams/9c05fab0-2d28-4094-a79e-2b37f8684fc3/content. Accessed 6 Jul 2026. 35579557

[25] Bachner F, Bobek J, Habimana K, Ladurner J, Lepuschütz L, Ostermann H, et al. Austria: health system review. 2018. https://iris.who.int/server/api/core/bitstreams/7451eef0-f7e1-4079-92d5-71b2abf19ae2/content. Accessed 6 Jul 2026. 30277215

[26] Economou C, Kaitelidou D, Karanikolos M, Maresso A, Greece. Health system review. 2017. https://iris.who.int/server/api/core/bitstreams/6547c59e-d2ab-4c5b-8dd6-40beec6d32e4/content. Accessed 6 Jul 2026. 29972131

[27] Des Jarlais DC, Lyles C, Crepaz N. Improving the reporting quality of nonrandomized evaluations of behavioral and public health interventions: the TREND statement. Am J Public Health. 2004;94:361–6. 10.2105/AJPH.94.3.361. PMC1448256 14998794

[28] Del Coronado-Vázquez MV, Gómez-Trenado R, Benito-Sánchez B, Barrio-Cortes J, Gil-Salmerón A, Amengual-Pliego M, Grabovac I. Cancer prevention in people experiencing homelessness: ethical considerations and experiences from the CANCERLESS project. Front Public Health. 2024;12:1371505. 10.3389/fpubh.2024.1371505. PMC11036339 38655508

[29] Evans SR. Clinical trial structures. J Exp Stroke Transl Med. 2010;3:8–18. 10.6030/1939-067x-3.1.8. PMC3059315 21423788

[30] FEANTSA. ETHOS: European Typology of Homelessness and Housing Exclusion. 2017. https://www.feantsa.org/download/ethos2484215748748239888.pdf. Accessed 17 Dec 2024.

[31] Belogianni K, Gil-Salmerón A, Fragner T, Gawronska J, Gomez RT, Tabaki I, et al. Feasibility and implementation insights from implementing the health navigator model for cancer prevention among people experiencing homelessness across Europe. Eur J Public Health. in press. 10.1093/eurpub/ckag119 PMC13470432 42592909

[32] EuroQol Research Foundation. EQ-5D-5L user guide. 2019. https://euroqol.org/publications/user-guides/. Accessed 19 Dec 2024.

[33] Yfantopoulos J. The Greek version of the EuropQol (EQ-5D) instrument. Archives Hellenic Med. 2001;18:180–91.

[34] Kontodimopoulos N, Pappa E, Niakas D, Yfantopoulos J, Dimitrakaki C, Tountas Y. Validity of the EuroQoL (EQ-5D) instrument in a Greek general population. Value Health. 2008;11:1162–9. 10.1111/j.1524-4733.2008.00356.x. 18489492

[35] Rowen D, Mukuria C, McDool E. A Systematic Review of the Methodologies and Modelling Approaches Used to Generate International EQ-5D-5L Value Sets. PharmacoEconomics. 2022;40:863–82. 10.1007/s40273-022-01159-1. 35829931

[36] Chew LD, Bradley KA, Boyko EJ. Brief questions to identify patients with inadequate health literacy. Fam Med. 2004;36:588–94. 15343421

[37] World Health Organization. The World Health Organization quality of life (WHOQOL) - BREF. 2004. https://iris.who.int/handle/10665/77773. Accessed 19 Dec 2024.

[38] Lloyd H, Fosh B, Whalley B, Byng R, Close J. Validation of the person-centred coordinated care experience questionnaire (P3CEQ). Int J Qual Health Care. 2019;31:506–12. 10.1093/intqhc/mzy212. PMC6839368 30508089

[39] Blasco-Calafat A, Blanes-Selva V, Fragner T, Doñate-Martínez A, Alhambra-Borrás T, García-Gómez JM, et al. Multi-source coherence analysis of the first European multi-centre cohort study for cancer prevention in people experiencing homelessness: a data quality study. medRxiv. 2024. 10.1101/2024.10.07.24314994. PMC12663700 41236789

[40] Blanca MJ, Arnau J, García-Castro FJ, Alarcón R, Bono R. Non-normal Data in Repeated Measures ANOVA: Impact on Type I Error and Power. Psicothema. 2023;35:21–9. 10.7334/psicothema2022.292. 36695847

[41] JASP Team. JASP (Version 0.19.3) [Computer software]; 2024.

[42] Brown J, Kaufman RE, Ariail M, Williams DL. Assessing patient navigation as a tool to address equity in cancer early detection. Transl Cancer Res. 2019;8:S421–30. 10.21037/tcr.2019.05.26. PMC8799019 35117118

[43] Dalton M, Holzman E, Erwin E, Michelen S, Rositch AF, Kumar S, et al. Patient navigation services for cancer care in low-and middle-income countries: A scoping review. PLoS ONE. 2019;14:e0223537. 10.1371/journal.pone.0223537. PMC6797131 31622363

[44] Sastry S, Zoller HM, Walker T, Sunderland S. From Patient Navigation to Cancer Justice: Toward a Culture-Centered, Community-Owned Intervention Addressing Disparities in Cancer Prevention. Front Commun. 2017. 10.3389/fcomm.2017.00019.

[45] Padgett DK. Homelessness, housing instability and mental health: making the connections. BJPsych Bull. 2020;44:197–201. 10.1192/bjb.2020.49. PMC7525583 32538335

[46] Teggart K, Neil-Sztramko SE, Nadarajah A, Wang A, Moore C, Carter N, et al. Effectiveness of system navigation programs linking primary care with community-based health and social services: a systematic review. BMC Health Serv Res. 2023;23:450. 10.1186/s12913-023-09424-5. PMC10165767 37158878

[47] Moravac CC. Reflections of Homeless Women and Women with Mental Health Challenges on Breast and Cervical Cancer Screening Decisions: Power, Trust, and Communication with Care Providers. Front Public Health. 2018;6:30. 10.3389/fpubh.2018.00030. PMC5863503 29600243

[48] Konstantinidis NV, Talias MA. Healthcare Professionals’ Perspectives on the Implementation of the Integrated Care Models for Chronic Patients. Cureus. 2023;15:e42173. 10.7759/cureus.42173. PMC10439480 37602035

[49] Calloo GV, Odei Boateng M, Agbe EA, Boateng GO. Shelter to Survival: Unpacking the Health Impacts of Housing Insecurity Across the Life Course. Int J Environ Res Public Health. 2026. 10.3390/ijerph23010091. PMC12840798 41595886

[50] Asgary R. Cancer care and treatment during homelessness. Lancet Oncol. 2024;25:e84–90. 10.1016/S1470-2045(23)00567-3. 38301706

[51] Rothenbühler M, Voorpostel M. Attrition in the Swiss Household Panel: Are Vulnerable Groups more Affected than Others? In: Oris M, Roberts C, Joye D, Ernst Stähli M, editors. Surveying Human Vulnerabilities across the Life Course. Cham: Springer International Publishing; 2016. pp. 223–44. 10.1007/978-3-319-24157-9_10.

