# Supplementary material for: Empowerment and quality of life through cancer prevention in people experiencing homelessness: evaluating the Health Navigator Model
Source: Health Qual Life Outcomes. 2026 Jul 19;24:131. doi: 10.1186/s12955-026-02588-8 (PMC13670686; doi:10.1186/s12955-026-02588-8)
Supplement: Supplementary file 1 [file 12955_2026_2588_MOESM1_ESM.docx]

|  | | **Healthcare empowerment (HCEQ)** | | | | | | | **HRQoL (EQ-5D-5L index)** | | | | | | | **Self-rated health (EQ VAS)** | | | | | | |
| --- | --- | --- | --- | --- | --- | --- | --- | --- | --- | --- | --- | --- | --- | --- | --- | --- | --- | --- | --- | --- | --- | --- |
|  | |  | **95% CI for mean difference** | |  | | | |  | **95% CI for mean difference** | |  | | | |  | **95% CI for mean difference** | |  | | | |
| **Comparison** | **Location** | **Mean difference** | **Lower** | **Upper** | **SE** | **df** | **Cohen’s d** | **p_bonf_** | **Mean difference** | **Lower** | **Upper** | **SE** | **df** | **Cohen’s d** | **p_bonf_** | **Mean difference** | **Lower** | **Upper** | **SE** | **df** | **Cohen’s d** | **p_bonf_** |
| **Baseline between countries** | Austria – Greece | 1.987 | 0.471 | 3.504 | 0.480 | 252 | 0.661 | 0.001 | 0.073 | -0.036 | 0.181 | 0.034 | 273 | 0.337 | 0.989 | -3.484 | -14.444 | 7.476 | 3.474 | 270 | -0.166 | 1.000 |
|  | Austria – Spain | 1.280 | -0.762 | 3.321 | 0.647 | 252 | 0.425 | 1.000 | 0.117 | -0.029 | 0.263 | 0.046 | 273 | 0.544 | 0.331 | 15.601 | 0.782 | 30.420 | 4.697 | 270 | 0.744 | 0.029 |
|  | Austria – UK | -1.309 | -2.996 | 0.378 | 0.534 | 252 | -0.435 | 0.418 | 0.204 | 0.082 | 0.326 | 0.039 | 273 | 0.946 | < .001 | 17.973 | 5.676 | 30.269 | 3.897 | 270 | 0.857 | < .001 |
|  | Greece – Spain | -0.708 | -2.605 | 1.190 | 0.601 | 252 | -0.235 | 1.000 | 0.044 | -0.091 | 0.180 | 0.043 | 273 | 0.206 | 1.000 | 19.085 | 5.398 | 32.772 | 4.338 | 270 | 0.910 | < .001 |
|  | Greece – UK | -3.296 | -4.805 | -1.787 | 0.478 | 252 | -1.096 | < .001 | 0.131 | 0.022 | 0.240 | 0.035 | 273 | 0.609 | 0.005 | 21.457 | 10.550 | 32.363 | 3.457 | 270 | 1.023 | < .001 |
|  | Spain – UK | -2.589 | -4.625 | -0.553 | 0.645 | 252 | -0.861 | 0.002 | 0.087 | -0.059 | 0.233 | 0.046 | 273 | 0.403 | 1.000 | 2.371 | -12.408 | 17.151 | 4.684 | 270 | 0.113 | 1.000 |
| **Follow-up between countries** | Austria – Greece | 3.870 | 2.307 | 5.434 | 0.495 | 252 | 1.287 | < .001 | 0.135 | 0.032 | 0.238 | 0.033 | 273 | 0.625 | 0.001 | 6.556 | -3.225 | 16.337 | 3.100 | 270 | 0.313 | 0.990 |
|  | Austria – Spain | 0.639 | -1.466 | 2.743 | 0.667 | 252 | 0.212 | 1.000 | 0.148 | 0.010 | 0.287 | 0.044 | 273 | 0.688 | 0.023 | 22.369 | 9.145 | 35.594 | 4.192 | 270 | 1.066 | < .001 |
|  | Austria – UK | -0.315 | -2.053 | 1.424 | 0.551 | 252 | -0.105 | 1.000 | 0.182 | 0.066 | 0.297 | 0.037 | 273 | 0.843 | < .001 | 18.689 | 7.715 | 29.663 | 3.478 | 270 | 0.891 | < .001 |
|  | Greece – Spain | -3.231 | -5.187 | -1.276 | 0.619 | 252 | -1.074 | < .001 | 0.013 | -0.115 | 0.142 | 0.041 | 273 | 0.062 | 1.000 | 15.813 | 3.599 | 28.028 | 3.871 | 270 | 0.754 | 0.002 |
|  | Greece – UK | -4.185 | -5.740 | -2.629 | 0.493 | 252 | -1.391 | < .001 | 0.047 | -0.056 | 0.150 | 0.033 | 273 | 0.218 | 1.000 | 12.133 | 2.400 | 21.866 | 3.085 | 270 | 0.578 | 0.003 |
|  | Spain – UK | -0.953 | -3.052 | 1.145 | 0.665 | 252 | -0.317 | 1.000 | 0.034 | -0.105 | 0.172 | 0.044 | 273 | 0.156 | 1.000 | -3.681 | -16.870 | 9.509 | 4.180 | 270 | -0.175 | 1.000 |
| **Baseline vs. follow-up within country** | Austria | -1.762 | -2.839 | -0.686 | 0.341 | 252 | -0.586 | < .001 | -0.040 | -0.104 | 0.023 | 0.020 | 273 | -0.187 | 1.000 | -9.563 | -17.098 | -2.027 | 2.388 | 270 | -0.456 | 0.002 |
|  | Greece | 0.121 | -0.716 | 0.957 | 0.265 | 252 | 0.04 | 1.000 | 0.022 | -0.027 | 0.071 | 0.016 | 273 | 0.101 | 1.000 | 0.477 | -5.245 | 6.200 | 1.814 | 270 | 0.023 | 1.000 |
|  | Spain | -2.403 | -3.890 | -0.917 | 0.471 | 252 | -0.799 | < .001 | -0.009 | -0.097 | 0.078 | 0.028 | 273 | -0.043 | 1.000 | -2.794 | -13.133 | 7.545 | 3.277 | 270 | -0.133 | 1.000 |
|  | UK | -0.768 | -1.836 | 0.300 | 0.338 | 252 | -0.255 | 0.673 | -0.062 | -0.127 | 0.002 |  | 273 | -0.290 | 0.067 | -8.846 | -16.324 | -1.368 | 2.370 | 270 | -0.422 | 0.006 |

***Table S1:*** Post hoc comparisons of primary outcomes. CI: Confidence interval; HCEQ: Healthcare empowerment questionnaire; UK: United Kingdom; VAS: Visual analogue scale.

***Fig. S1:*** Raincloud plots of country-specific primary outcome scores over time.
